# Recurrence of Merkel cell carcinoma in the gastrointestinal tract: a case report

**DOI:** 10.1186/s13104-015-1139-3

**Published:** 2015-05-07

**Authors:** Rasikh Tuktamyshov, Dhanpat Jain, Philip M Ginsburg

**Affiliations:** Yale New Haven Hospital, 1450 Chapel Street, New Haven, CT 06511 USA; Department of Pathology, Yale University, 310 Cedar Street, New Haven, 208023 USA; Yale-New Haven Hospital, 2200 Whitney Avenue, Suite 360, Hamden, New Haven, CT 06518 USA

**Keywords:** Merkel Cell carcinoma, Polyp, Colonoscopy, Colon, Metastasis

## Abstract

**Background:**

Merkel cell carcinoma is a rare and aggressive skin malignancy that arises from primary neural cells and has a tendency for local recurrence and regional lymph node metastases. There are only a few cases in the literature reporting metastases of Merkel cell carcinoma to the gastrointestinal tract.

**Case presentation:**

We present a 70 year old Caucasian female with distant history of Merkel cell carcinoma who presented with iron-deficiency anemia. Colonoscopy performed later for the evaluation of anemia revealed 1 cm polyp in ascending colon which turned out to be the recurrence of Merkel cell carcinoma.

**Conclusion:**

Metastatic Merkel cell carcinoma to the gastrointestinal tract or any other organ should be considered in patients with a history of Merkel cell carcinoma.

## Background

Merkel cell carcinoma (MCC) is a rare condition. Earlier studies suggested that MCC originates from Merkel cells [1] which are mechanoreceptors located at stratum basale of the epidermis [2]. However, MCC probably arises from some kind of progenitor cell of the skin as substantial differences exist between MCC and Merkel cells [3]. Clinically, patients with MCC typically present with a rapidly growing, painless, firm, nontender, shiny, flesh-colored or bluish-red, intracutaneous nodule [1,4]. After resection, local recurrence is common but a review of the literature found only a few cases of documented metastases to gastrointestinal (GI) tract [4]. We present a case of Merkel cell carcinoma of the skin that recurred in the colon.

### Case Presentation

A 70-year-old Caucasian female was evaluated for a one month history of progressively worsening low back pain and weakness. She had no gastrointestinal tract complaints. Her past medical history was significant for multiple basal and squamous cell cancers, as well as Merkel cell carcinoma 5 years previously, at which time she presented with a lump in her left groin. After local resection, she was treated with radiation and carboplatin. There was no recurrence and a subsequent abdominal CT (computed tomography) scan 3 years later showed no evidence of metastatic disease. The primary site was thought to be an inguinal lymph node.

**Figure 1:**
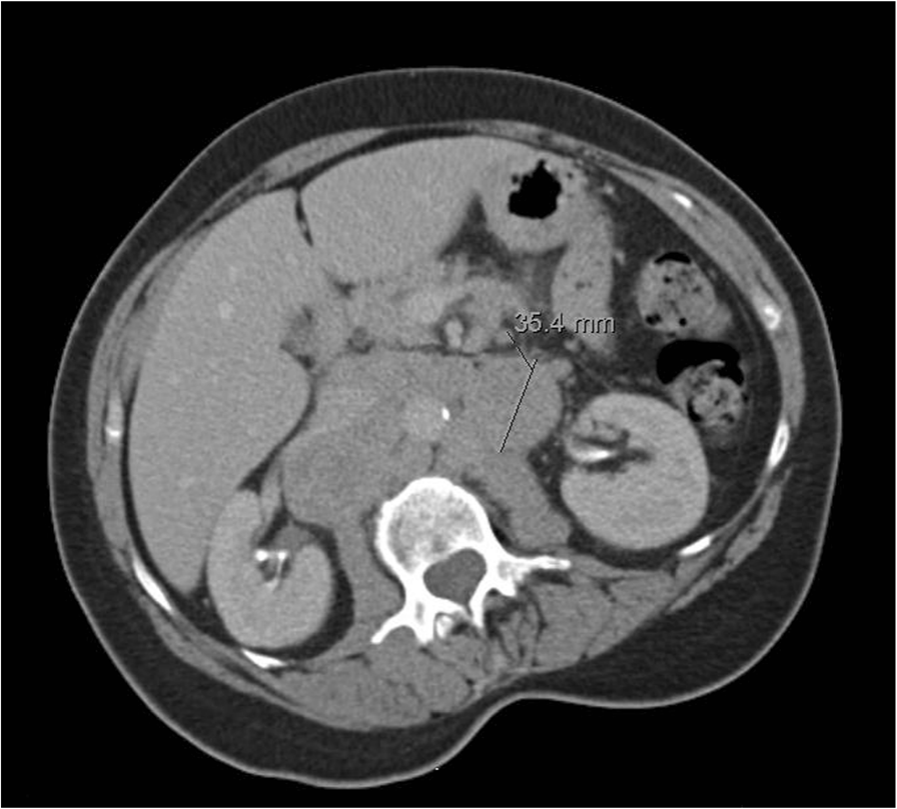
CT scan. Extensive bulky retroperitoneal lymphadenopathy (lymph node of 35.4 mm is marked on the picture).

On physical exam she had a 5 mm mobile lymph node in the left supraclavicular area. There were no focal neurological deficits. Stool tested guaiac negative. Labs revealed hemoglobin 7.1 g/dL, hematocrit 21.1%, iron level 15 mcg/dL, total iron binding capacity 330 mg/dL, and transferrin saturation 5%, consistent with severe iron-deficiency anemia. A contrast enhanced abdominal CT scan showed bulky retroperitoneal adenopathy encompassing the renal arteries, aorta, inferior vena cava as well as circumferentially thickened loop of small bowel in the left hemiabdomen (Figure 1). A small bowel series was negative. The decision was made to perform a colonoscopy.

**Figure 2:**
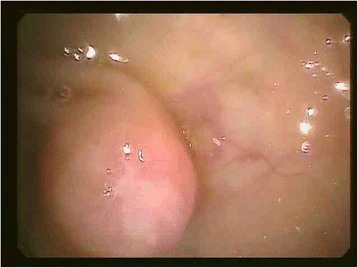
Colonoscopy, 10 mm sessile polyp.

**Figure 3:**
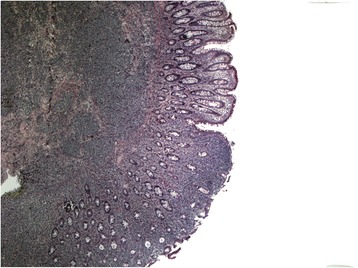
Low magnification photograph showing the tumor infiltrating into the submucosa as well as overlying mucosa. The tumor cells are diffusely infiltrating the tissues without any specific arrangement or pattern.

**Figure 4:**
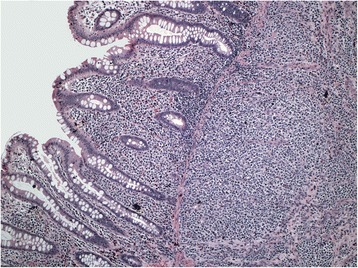
Higher magnification to show tumor cells that are small with hyperchromatic nuclei and scanty cytoplasm.

**Figure 5:**
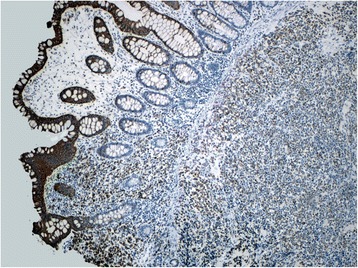
Immunostain for cytokeratin 20 showing diffuse cytoplasmic and surface positivity.

**Figure 6:**
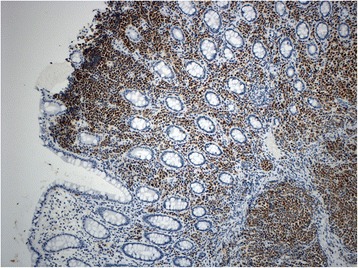
Immunostain for endocrine markers chromogranin and synaptophysin were similar and showed strong cytoplasmic positivity.

The findings at colonoscopy (Figure 2) revealed a semi-sessile ascending colon polyp measuring approximately 1 cm in size. The polyp was smooth and pale, with regular margins, and normal surrounding mucosa. It was resected as a single piece using hot snare polypectomy technique, and all tissue was recovered into a sterile trap and submitted for routine histology. Post-resection appearances suggested complete removal of all polypoid mucosa. Pathological examination of the specimen showed a small blue cell neoplasm involving both the mucosa and submucosa (Figures 3, 4, 5 and 6). The tumor cells were relatively small with scanty cytoplasm and hyperchromatic round nuclei. Immunostain for cytokeratin 20 showing perinuclear dot like cytoplasmic and surface positivity. Immunostaining for endocrine markers chromogranin and synaptophysin showed strong cytoplasmic positivity. The findings are typical of MCC and were similar to the patient’s previous groin tumor. The patient was diagnosed with stage IV MCC based on the presence of a distant metastasis.

The patient was treated with 4 cycles of cisplatin and etoposide (VP-16). Five months later, a CT of the abdomen showed increase in abdominal lymphadenopathy and a midjejunal small bowel mass that was felt to represent a metastatic focus. She received an additional 8 cycles of chemotherapy with cyclophosphamide, doxorubicin and vincristine and a follow-up positron emission tomography scan showed resolution of the previous multifocal lymphadenopathy in the chest, abdomen, and pelvis with no new lesions.

## Discussion

Merkel cell cancer is a rare skin malignancy. In the Surveillance, Epidemiology, and End Results (SEER) database a total of 1665 cases were identified between 1973 and 2002 [5]. The cause is unknown. Sunlight has been implicated as a causative factor, in view of its predilection for sun-exposed regions of the body and geographic distribution [1,2]. In one prospective study, the highest age adjusted incidence among a Caucasian population was in Hawaii, the geographic location with the highest ultraviolet B index [6]. Immunosuppression has been associated with MCC, and linked with organ transplantation [7]. Cases of MCC were reported among patients taking immunosuppressing medications like tumor necrosis factor inhibitors, including etanercept (Enbrel®) [8] and adalimumab (Humira®) [9]. In 2008, a previously unknown polyomavirus was detected in 80% of patients with MCC in a series of 10 patients, suggesting a possible role in the pathogenesis [10].

On light microscopy MCC may be confused with poorly differentiated small-cell tumors such as small-cell lung cancer, lymphoma, neuroblastoma and other “small round blue cell tumors”, which are characterized by a superficially very blue appearance on routine H & E stained sections due to the cells having a single large hyperchromatic nucleus with evenly distributed granular chromatin and a thin rim of cytoplasm [11]. This appearance is shared by many tumors including small cell carcinoma, lymphoma, Ewing sarcoma and rhabdomyosarcoma, amongst others. The pathologic differential diagnosis of Merkel cell carcinoma includes other small round blue cell tumors, particularly lymphoma and small cell carcinoma when encountered in the GI tract. The immunohistochemical profile of the tumor is very typical with perinuclear punctate or dot-like positivity for CK20 (cytokeratin 20) and endocrine markers. This is in contrast to small cell carcinomas which, although frequently positive for endocrine markers, are negative for CK20. The small cells carcinomas in addition often show positivity of TTF-1 (thyroid transcription factor-1) or less commonly CDX-2 (caudal homeobox 2 protein) in the GI tract. Lymphomas can be easily differentiated by positivity for various leukocyte markers and lack of keratins and endocrine markers.

Our case report presents a patient with MCC metastatic to colon. The jejunal mass and lymphadenopathy seen on the initial abdominal CT before the colonoscopy were most likely metastasis of MCC but not confirmed by tissue biopsy. The patient responded well to chemotherapy.

The patient in our case had nodal disease without an apparent primary site, and this was the case for about 2% of initial presentations out of 661 cases published by Tai *et al.* [12]*.* The patient also had previous basal and squamous cell carcinomas of the skin. The risk of development of MCC is increased in patients with other malignancies including skin cancers and melanoma. Moreover, MCC is a risk factor for development of another primary cancer with the highest risk for salivary gland tumors, bladder cancer, and multiple myeloma [13].

There are only a few cases in the literature reporting MCC metastases to the GI tract [14-19] and only three cases to our knowledge with metastasis to the large intestine. One of them describes a case with metastases to the colon, duodenum and stomach 6 years after the excision of a skin lesion on the neck that was initially diagnosed as basal cell carcinoma but reevaluation of the specimen confirmed MCC [14]. The second one reports the case of a patient who presented with hematochezia from MCC in the rectum [15]. A third case reports primary head and neck MCC with metastasis to the colon who survived for 6 years [16].

There are more cases that report MCC metastatic to other parts of GI tract. One of them describes a patient with acute upper GI bleeding from a gastric metastasis [17]. Krasagakis *s*. presented the case of a patient who had widely spread metastatic disease including the stomach [18]. Another case presented the recurrence of MCC at ileocecal valve 18 months after resection of a tumor on the neck [19].

## Conclusion

MCC is a rare disease that rarely metastasizes to the gastrointestinal tract and may mimic other small blue round cell tumors. The morphology combined with immunostaining profile helps to establish the correct diagnosis. Metastatic disease should be in the differential diagnosis when evaluating GI symptoms in a patient with a history of MCC.

### Consent

Written informed consent was obtained from the patient's next-of-kin for publication of this case report and any accompanying images. A copy of the written consent is available for review by the Editor-in-Chief of this journal.

## Abbreviations

MCC: Merkel cell carcinoma
GI: Gastrointestinal tract
CT: Computed tomography
CK20: Cytokeratin 20
TTF-1: Thyroid transcription factor-1
CDX-2: Caudal homeobox 2 protein
